# Comparative evaluation of sequencing platforms: Pacific Biosciences, Oxford Nanopore Technologies, and Illumina for 16S rRNA-based soil microbiome profiling

**DOI:** 10.3389/fmicb.2025.1633360

**Published:** 2025-08-06

**Authors:** Vladimir Veselovsky, Mikhail Romanov, Polina Zoruk, Andrey Larin, Vladislav Babenko, Maxim Morozov, Aleksandra Strokach, Natalia Zakharevich, Sadokatkhon Khamidova, Albina Danilova, Aleksey Vatlin, Vsevolod Pavshintsev, Feng Chenguang, Denis Tsybizov, Nikita Mitkin, Olesya Galanova, Alexander Zakharenko, Kirill Golokhvast, Ksenia Klimina

**Affiliations:** ^1^Lopukhin Federal Research and Clinical Center of Physical-Chemical Medicine of Federal Medical Biological Agency, Moscow, Russia; ^2^Siberian Federal Research Center of Agrobiotechnology RAS, Krasnoobsk, Russia; ^3^Institute of Ecology, Peoples’ Friendship University of Russia (RUDN University), Moscow, Russia; ^4^Collagel LLC, Moscow, Russia; ^5^Moscow Center for Advanced Studies, Moscow, Russia; ^6^Advanced Engineering School “Agrobiotek” of the Tomsk State University, Tomsk, Russia

**Keywords:** environmental sample, full-length 16S rRNA sequencing, V3–V4 and V4 regionsPacBio, Oxford Nanopore, Illumina, soil microbiome, sequencing platform comparison

## Abstract

Soil microbiome profiling is crucial for understanding microbial diversity and its roles in ecosystem functioning and agricultural productivity. Recent advancements in high-throughput sequencing, particularly Pacific Biosciences (PacBio) and Oxford Nanopore Technologies (ONT), offer long-read sequencing capabilities that enhance microbial community analysis. In this study, we performed a comparative evaluation of 16S rRNA gene sequencing using Illumina (V4 and V3–V4 regions), PacBio (full-length and trimmed V3–V4/V4 regions), and ONT (full-length) to assess bacterial diversity in soil microbiomes. We analyzed three distinct soil types and applied standardized bioinformatics pipelines tailored to each platform. To ensure comparability, sequencing depth was normalized across platforms (10,000, 20,000, 25,000, and 35,000 reads per sample). Our results demonstrated that ONT and PacBio provided comparable bacterial diversity assessments, with PacBio showing slightly higher efficiency in detecting low-abundance taxa. Despite differences in sequencing accuracy, ONT produced results that closely matched those of PacBio, suggesting that ONT’s inherent sequencing errors do not significantly affect the interpretation of well-represented taxa. Our study demonstrated that, regardless of the sequencing technology used and the choice of the target region (full-length 16S rRNA gene or its regions), microbial community analysis ensures clear clustering of samples based on soil type. The only exception is the V4 region, where no soil-type clustering is observed (*p* = 0.79). These results provide a comprehensive evaluation of sequencing platform performance.

## Introduction

Microbiome profiling is essential for understanding the complex living network within soil that plays an important role in agricultural systems (Mas-Carrió et al., 2018). It provides vital information on soil ecology (Chen et al., 2021) and helps in identifying microbiomes indicative of soil fertility and crop production, which is crucial for developing sustainable agriculture (Zhang et al., 2022).

Despite the importance of soil microbiome profiling, conventional techniques such as culturing and PCR-based methodologies face significant limitations. One major challenge is the high diversity and heterogeneity of soil microbial community (MC), which complicates the detection and monitoring of microbial responses to different management practices (Erlandson et al., 2024). Additionally, there is a lack of soil-specific reference databases available to classifiers, which hinders the accurate representation of the soil community complexity (Edwin et al., 2024).

Recent advancements in soil microbiome characterization have been driven by the development and application of high-throughput sequencing (HTS) techniques (Garg et al., 2024) which simplify description of MC composition in various habitats compared to traditional methods (Zhang G. et al., 2024; Zhang T. et al., 2024) and allow the development of modern indicators of soil biological quality (Djemiel et al., 2022). Traditional methods like culturing and Sanger sequencing are limited by biases and low throughput, often underrepresenting microbial diversity. HTS analyzes thousands to millions of DNA sequences in parallel, offering identification of both abundant and rare taxa, enabling assessment of functional potential and exploration of MC dynamics across spatial and temporal scales. Additionally, HTS reduces time and cost by automating processes, providing rapid, large-scale analysis of soil ecosystems, crucial for studying biogeochemical cycles, plant-microbe interactions, and environmental responses (Caporaso et al., 2012).

Amplicon sequencing of the 16S ribosomal RNA (16S rRNA) gene is considered a reliable and efficient method for taxonomic classification of MC and analyzing their associated characteristics (Yarza et al., 2014). Third-generation sequencing solutions, represented by PacBio and ONT, appear to be superior platforms compared to traditional methods such as Illumina and Sanger sequencing due to their ability to produce long reads, allowing for full-length 16S rRNA gene sequencing, which offers finer taxonomic resolution (Garg et al., 2024). Traditional short-read methods (100–400 bp), such as Illumina, usually target hypervariable regions, e.g., V3–V4 (Klindworth et al., 2013), which can lead to ambiguous taxonomic assignments (Callahan et al., 2019). Long-read sequencing overcomes this limitation and improves species-level identification.

PacBio platform, utilizing the circular consensus sequencing (CCS) model, is capable of fully covering the complete 16S rRNA sequences, providing high-resolution species-level identification with an exceptional accuracy exceeding 99.9% (Johnson et al., 2019). The high accuracy of PacBio reads is achieved through multiple passes of the same DNA molecule, which offers detailed insights into specific genomic regions (Garg et al., 2024). The existing data on ONT use in 16S rRNA sequencing is more controversial due to higher error rates compared to PacBio (Zhang et al., 2023). Meanwhile, recent modifications of ONT, with the latest reagent kits, flow cells with double reader-head (particularly, R10.4.1 flow cell used in current study), and basecalling algorithms significantly improved the base accuracy to over 99% (Ermini and Driguez, 2024). Additionally, application of algorithms developed precisely for MC profiling from full-length 16S rRNA sequences, such as Emu, ensures generation of fewer false positives and false negatives than alternative methods, thus reducing the error rates (Curry et al., 2022). Furthermore, a recent study reported *Q*-scores close to *Q*28 (~99.84% base accuracy) for ONT reads, emphasizing the notable improvement in sequencing quality achieved by Nanopore platforms in recent years (Zhang G. et al., 2024; Zhang T. et al., 2024). This trend of increasing basecalling accuracy, coupled with improved library preparation protocols and real-time data processing, highlights the rapidly growing potential of ONT for accurate and large-scale metataxonomic studies.

In recent years, both PacBio and ONT sequencing platforms have significantly improved in accuracy, enabling more precise analysis of MC. However, challenges remain, such as the higher error rates inherent to ONT reads and the reliance on error-correction algorithms in PacBio sequencing (Zhang et al., 2023).

Although comparative studies of microbiome sequencing data, including soil microbiomes, have been conducted, they typically focus on pairwise platform comparisons and often lack biological replication (Stevens et al., 2023; Yeo et al., 2024; Biada et al., 2025). In contrast, our study includes three independent biological replicates per soil sample, enabling a more robust comparison of three sequencing technologies (Illumina, PacBio, ONT) and minimizing random variation. This approach enhances the reliability of diversity estimates and strengthens conclusions regarding the comparative performance of the platforms. However, few studies have evaluated the combined impact of sequencing technology, read depth, and analytical tools on soil microbiota profiling using full-length 16S rRNA gene sequencing. Our work addresses this gap by directly comparing all three platforms under controlled conditions, incorporating multiple sequencing depths and a standardized bioinformatic pipeline.

Building on this experimental design, we systematically assessed the performance of PacBio, ONT, and Illumina platforms in detecting bacterial diversity in soil samples. By analyzing full-length 16S rRNA gene sequences across different read depths (10,000, 20,000, 25,000, and 35,000 reads per sample), we compared alpha and beta diversity metrics and examined the taxonomic resolution of each platform. This study provides new insights into the relative strengths and limitations of each technology for soil microbiome research.

## Materials and methods

### Soil samples collection

The size of the experimental plots was 15 m × 18 m, the experiment was located in 3-fold replication. Soil samples were collected at the 0–10 and 10–20 cm soil layers. The sample for analysis was an average sample of 5 individual ones, collected at each spatial replication of the experiment, with three replications of the experiment. On the long-term fallow, soil sampling was carried out according to the same scheme. Soil samples were collected from medium-humus, medium-loamy chernozem (Luvic Chernozem) in the forest-steppe region of the Ob area (coordinates: 54° 53′13.5″ N, 82° 59′36.7″E). The soil samples were delivered to the laboratory, passed through a sieve with a cell diameter of 1 mm under sterile conditions, placed in sterile containers and stored at −20°C until DNA extraction. Thus, the pattern of obtaining samples for each soil type: 2 layers, 3 replications.

### DNA extraction

Samples were homogenized and then DNA was extracted using the Quick-DNA Fecal/Soil Microbe Microprep kit (Zymo Research, United States) following the manufacturer’s protocol. The extracted DNA was quantified using a Qubit 4 Fluorometer (Thermo Fisher Scientific, United States) and quality was assessed by electrophoresis in 1% agarose gel. ZymoBIOMICS Gut Microbiome Standard (D6331) was extracted using the same protocol.

### 16S rRNA gene sequencing on the PacBio platform

The full-length 16S rRNA gene was amplified from 5 ng of genomic DNA using the universal primers 5′-GCATC/barcode/AGRGTTYGATYMTGGCTCAG-3′ and 5′-GCATC/barcode/RGYTACCTTGTTACGACTT-3′ (PN 101-599-700, PacBio Protocol), each tagged with sample-specific PacBio barcodes for multiplexed sequencing. PCR amplification was performed over 30 cycles: denaturation at 95°C for 30 s, annealing at 57°C for 30 s, and extension at 72°C for 60 s. Post-PCR quality was assessed using a Fragment Analyzer (Agilent Technologies, United States), and equimolar DNA concentrations from each sample were pooled. Library preparation was conducted with the SMRTbell Prep Kit 3.0 (PacBio, United States) following PacBio’s 16S SMRTbell protocol. Library concentration and size were measured using the Qubit HS DNA Kit (Invitrogen, United States) and a Fragment Analyzer. Sequencing was performed on the PacBio Sequel IIe system with a run time of 10 h.

### 16S rRNA gene sequencing on the MinION platform

PCR amplification, library preparation, and sequencing on the MinION platform were performed as described in our previous work (Strokach et al., 2025). Briefly, the 16S rRNA gene was amplified using the primers 27F (AGAGTTTGATYMTGGCTCAG) and 1492R (GGTTACCTTGTTAYGACTT), and amplicons were purified with KAPA HyperPure Beads (Roche, Switzerland). Libraries were prepared using the Native Barcoding Kit 96 (SQK-NBD109.96) and sequenced on an R10.4.1 flow cell (FLO-MIN114) with the MinION Mk1B device. Sequencing data were acquired using MINKNOW software (ver. 24.06.14). Basecalling was performed using dorado (ver. 7.6.7) (dorado) with hac model and Phred score >7.

### 16S rRNA gene sequencing on the Illumina platform

PCR amplification, library preparation, and sequencing on the Illumina platform were performed as described in our previous work (Larin et al., 2024). The V3–V4 region was amplified using primers incorporating Illumina adapter sequences: forward (TCGTCGGCAGCGTCAGATGTGTATAAGAGACAGCCTACGGGNGGCWGCAG) and reverse (GTCTCGTGGGCTCGGAGATGTGTATAAGAGACAGGACTACH VGGGTATCTAATCC) (Klindworth et al., 2013). For the V4 region, the primers 515F (TCGTCGGCAGCGTCAGATGTGTATAAGAGACAGGTGBCAGC MGCCGCGGTAA) and 805R (GTCTCGTGGGCTCGGAGATGTGTATAAGAGACAGGACTACN VGGGTMTCTAATCC) were used. Amplification was conducted using the Tersus Plus PCR Kit (Evrogen, Russia), followed by indexing with Illumina dual indices. Library quality and size distribution were assessed using a high-sensitivity DNA chip (Agilent Technologies), and quantification was performed with the Quant-iT DNA Assay Kit, High Sensitivity (Thermo Fisher Scientific, United States). Sequencing was carried out on the MiSeq platform (Illumina, United States) using the MiSeq Reagent Kit v2 (500 cycles) and 20% Phix.

### Bioinformatics and statistical analysis 16S rRNA data from ONT

Raw ONT reads were downsampled to 10,000, 20,000, 25,000 and 35,000 reads using the Python Bio package. Adapter sequences were removed using Porechop (ver. 0.2.4)^1^ with default parameters. Reads were filtered using Chopper (ver. 0.6.0) (De Coster and Rademakers, 2023) with the parameters -l 1,300 and --maxlength 1,600, removing sequences with a Phred score below 10 or outside the length range of 1,300–1,600 nucleotides. Taxonomic classification was performed using the Emu pipeline (ver. 3.4.5) (Curry et al., 2022), which analyzed the processed sequences. Additionally, NanoStat (ver. 1.6.0) (De Coster et al., 2018) was used to generate comprehensive quality and distribution statistics for the filtered reads. Processed data were imported into RStudio (ver. 2023.12.0 + 369, R ver. 4.3.2) for downstream analysis using the MicrobiotaProcess package (ver. 1.17.1) (Xu et al., 2023). Statistical significance between groups was determined using distance-based permutational multivariate analysis of variance (PERMANOVA) via the mp_adonis function, with Bray–Curtis distances and significance set at *p* < 0.05 based on 9,999 permutations. ZymoBIOMICS Gut Microbiome Standard were processed the same way as the soil ONT data except the initial step of rarefaction. Results of taxonomic annotation were imported into Rstudio for further analysis.

An additional database was created based on the Genome Taxonomy Database (GTDB) genus database by using emu build-database command from Emu pipeline (ver. 3.4.5). The created database was used for taxonomic classification with the same tools as in case with standard emu database for further comparison with results of analysis of Illumina V3–V4 region reads. To ensure taxonomic consistency across replicates, we applied an additional filtering step at the species level. Each soil sample was sequenced in triplicate, and only bacterial taxa detected in all three replicates of a given sample were retained.

### Bioinformatics analysis of full-length 16S rRNA data from PacBio

Demultiplexing of the sequencing reads was performed with PacBio lima (2.5.1). HiFi reads (CCS reads with a predicted accuracy ≥Q20) were extracted using SAMtools (1.13) (Danecek et al., 2021) and converted to FASTQ format using PacBio bam2fastq (1.3.1). Primers were trimmed from each read using cutadapt (4.2) (Martin, 2011). To enable direct comparison with ONT sequencing data, PacBio reads were downsampled to 10,000, 20,000, and 25,000 reads using the Python Bio package. These reads were processed using Chopper (ver. 0.6.0), Emu (ver. 3.4.5), and NanoStat (ver. 1.6.0) with the same parameters as those applied to ONT data. The resulting data were imported into RStudio (ver. 2023.12.0 + 369, R 4.3.2) and analyzed following the same workflow used for ONT data. The Procrustes test was applied to compare the taxonomic data structure of ONT and PacBio on every sequencing depth. The protest function with 999 permutations from vegan R package (ver. 2.6-6.1) was used to carry out this test.

### Bioinformatics analysis of V3–V4 and V4 regions of 16S rRNA data for PacBio

Cutadapt (ver. 4.9) (Martin, 2011) was used to extract specific 16S rRNA regions from full-length PacBio reads. For the V4 region, primer sequences 515F (GTGYCAGCMGCCGCGGTAA) and 805R (GGATTAGATACCCTGGTA) were applied with the following parameters:

-g “GTGYCAGCMGCCGCGGTAA; rightmost. GGATTAGATACCCTGGTA”
-e 0.2
--discard-untrimmed

Similarly, the V3–V4 region was extracted using primers 341F (CCTACGGGNGGCWGCAG) and 801R (GACTACHVGGGTATCTAATCC) with the same Cutadapt parameters. The extracted sequences were processed using the DADA2 pipeline (ver. 1.30.0) (Callahan et al., 2016) with the following settings: Filtering and trimming: filterAndTrim(truncLen = 0, maxN = 0, maxEE = 2, truncQ = 2, rm.phix = TRUE).

Chimera removal: removeBimeraDenovo(method = “consensus”).

Taxonomic classification was performed using the GTDB genus database and DADA2 R package.

### Bioinformatics analysis of 16S rRNA data for Illumina

Raw Illumina paired-end reads were processed using the fastp tool (ver. 0.22.0) (Chen et al., 2018) for quality control and adapter removal, with the --detect_adapter_for_pe option enabled. The adapter-free paired-end reads were then merged using the same tool with the --merge option. Further processing was conducted using the DADA2 pipeline (ver. 1.30.0) (Callahan et al., 2016) with GTDB genus database. To maintain consistency with PacBio V3–V4 and V4 datasets, 18,000 reads were randomly subsampled from the merged reads for downstream analysis. The bioinformatics workflow followed the same approach as for PacBio data, including taxonomic classification and microbial community diversity assessments.

### Comparative analysis of 16S rRNA gene sequencing using Illumina and ONT

Illumina V3–V4 region of 16S rRNA gene sequencing data were processed using the DADA2 pipeline, with taxonomic classification performed against the GTDB genus-level database (file GTDB_bac120_arc122_ssu_r202_Genus.fa.gz) (Alishum, 2021). For ONT full-length 16S rRNA sequencing data the Emu pipeline was used. We used the same database as for Illumina data and modified it to make it compatible with Emu to ensure consistency in taxonomic assignment. Bacterial taxa detected in all three replicates of a given sample were retained.

### Comparative analysis of sequencing technologies

**Figure 1:**
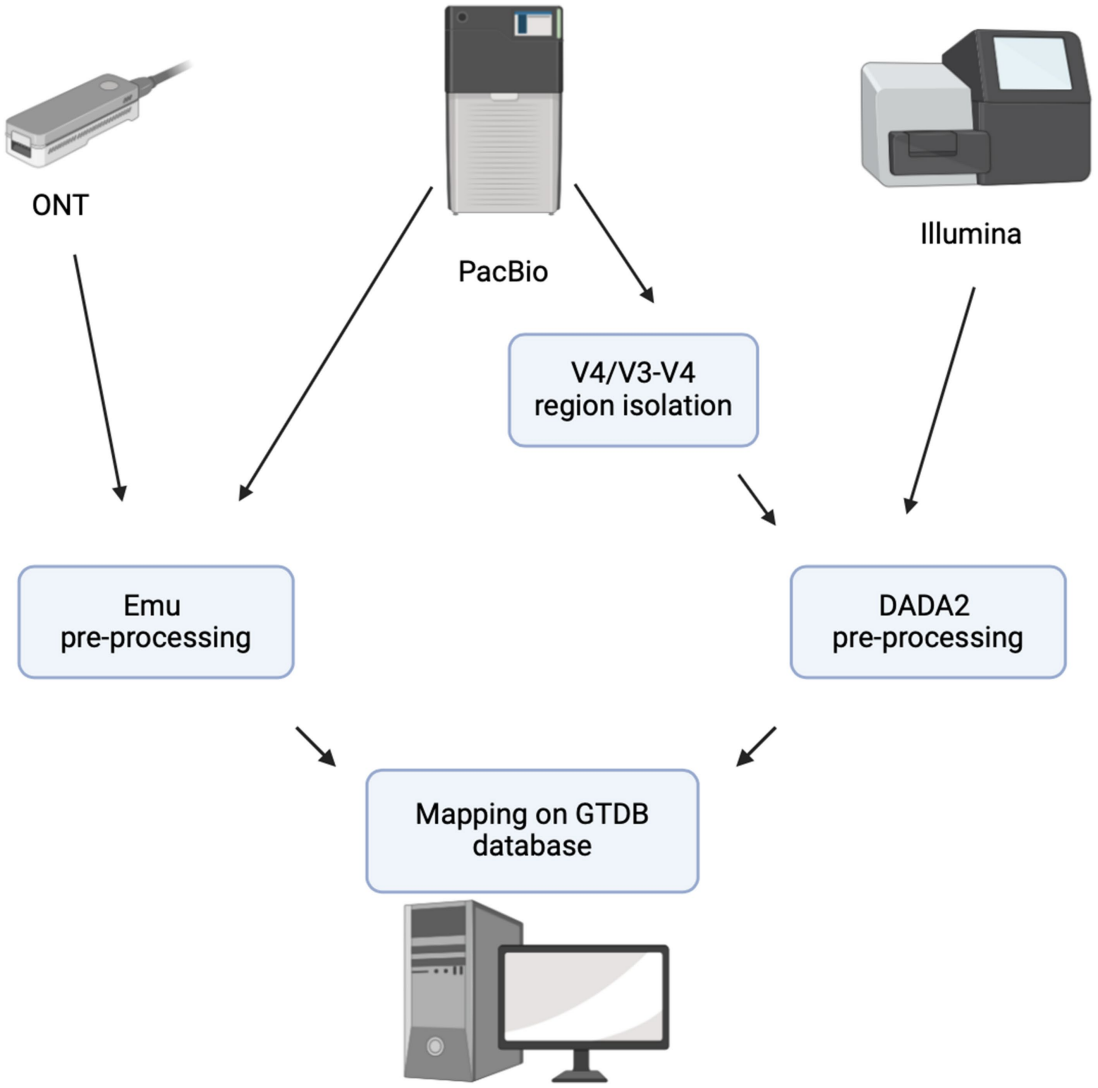
Data analysis workflow.

For cross-platform comparisons, all sequencing reads were preprocessed using platform-specific tools to ensure consistency. Emu (ver. 3.4.5) was used for ONT and PacBio full-length reads, while the DADA2 pipeline (ver. 1.30.0) with the GTDB genus database was applied to PacBio V4/V3–V4 and Illumina reads (Figure 1). This standardized approach allowed for a direct comparison of sequencing platforms in terms of taxonomic resolution and MC profiling.

All of the scripts we applied you can find via this link: https://github.com/mromanov2001/Comparative-Evaluation-of-Sequencing-Platformsfor-16S-rRNA-Based-Soil-Microbiome-Profiling.

The raw sequencing data obtained using PacBio, ONT and Illumina sequencing were submitted to the NCBI Sequence Read Archive (SRA) and are accessible under the following BioProject identifiers: PRJNA1190309 (for 16S rRNA gene sequencing on ONT), PRJNA1190314 [for 16S rRNA gene sequencing on Pacific Biosciences (PacBio)], PRJNA1190320 and PRJNA1190324 (for V3–V4 and V4 region of the 16S rRNA gene sequencing data on Illumina (MiSeq) respectively).

## Results

### PacBio vs. ONT: read depth affects the efficiency of species identification

We compared the performance of the PacBio and ONT sequencing platforms at varying read depths to analyze the MC in soil samples. The initial mean read count was 41,070 for PacBio and 83,617 for ONT. After filtering, the average read count remained almost unchanged for PacBio (40,856), while ONT experienced a more substantial reduction, retaining 69,896 reads on average. The percentage of reads filtered out was significantly lower for PacBio (0.52%) compared to ONT (16.7%), reflecting the higher sequencing error rate associated with ONT. Detailed information on the initial read counts for each sample is provided in Supplementary Table S1. To evaluate the effect of read depth on bacterial diversity detection, raw sequencing reads were downsampled to 10,000, 20,000, 25,000, and 35,000 per sample. The latest number of reads was chosen due to minimal reads presence among PacBio samples data. Since each sample was sequenced in three biological replicates, we applied an additional filtering criterion to ensure the reliability of detected bacterial taxa. Only bacterial species that were consistently present across all three replicates of a given sample were retained for further analysis. This approach minimizes the impact of random sequencing errors and rare artifacts, enhancing the robustness of taxonomic assignments and comparative assessments between sequencing platforms.

Increasing the number of reads per sample led to a significant rise in the number of identified species for both PacBio and ONT (Table 1). As the read count increased from 10,000 to 20,000, the number of identified species rose 1.58-fold for PacBio (348 to 549) and 1.7-fold for ONT (267 to 461). At 25,000 reads, species counts reached 1,16 for PacBio and 1,1 for ONT, further increasing to 1,23 and 1,3, respectively, at 35,000 reads (Table 1), while the average percentage of unclassified reads remained low, at 0.12% for PacBio and 0.04% for ONT (Supplementary Table S2). These results highlight the positive correlation between read depth and species identification. When analyzing the rarefaction curves, we observed that bacterial diversity in the samples reached a plateau after 35,000 reads, indicating sufficient taxonomic coverage (Supplementary Figure S1).

**Table 1:** The number of bacterial species identified from sequencing data obtained using PacBio and ONT platforms at different read depths.

Number of reads per sample	Number of species detected^a^		
	PacBio	ONT	Delta %
10,000	348	267	23.3
20,000	549	461	16
25,000	637	519	18.5
35,000	784	663	15.4

a Wilcoxon signed rank exact test shows no significant difference in number of identified species between PacBio and ONT platforms (*p*-value = 0.125).

To further evaluate the impact of ONT basecalling accuracy on downstream taxonomic profiling, we applied the Dorado basecaller in both high accuracy (HAC) and super-accuracy (SUP) models to ONT sequencing data from the ZymoBIOMICS Gut Microbiome Standard. Precision, recall, and *F*_1_-score were calculated, considering only bacterial species with a relative abundance greater than 0.5% as present. All metrics were identical for both basecalling modes: precision = 0.91, recall = 0.77, and *F*_1_-score = 0.83. The number of reads retained after filtering was also comparable between the two models: 39,323 for SUP and 38,423 for HAC (Supplementary Table S1). Moreover, the overall taxonomic composition of the standard was consistent with the manufacturer’s expected profile in both basecalling models, indicating reliable identification of dominant taxa regardless of the model used (Supplementary Table S3). It is important to note that the Dorado sup mode requires substantially more computational time and resources.

### Majority of species are common to both platforms

**Figure 2:**
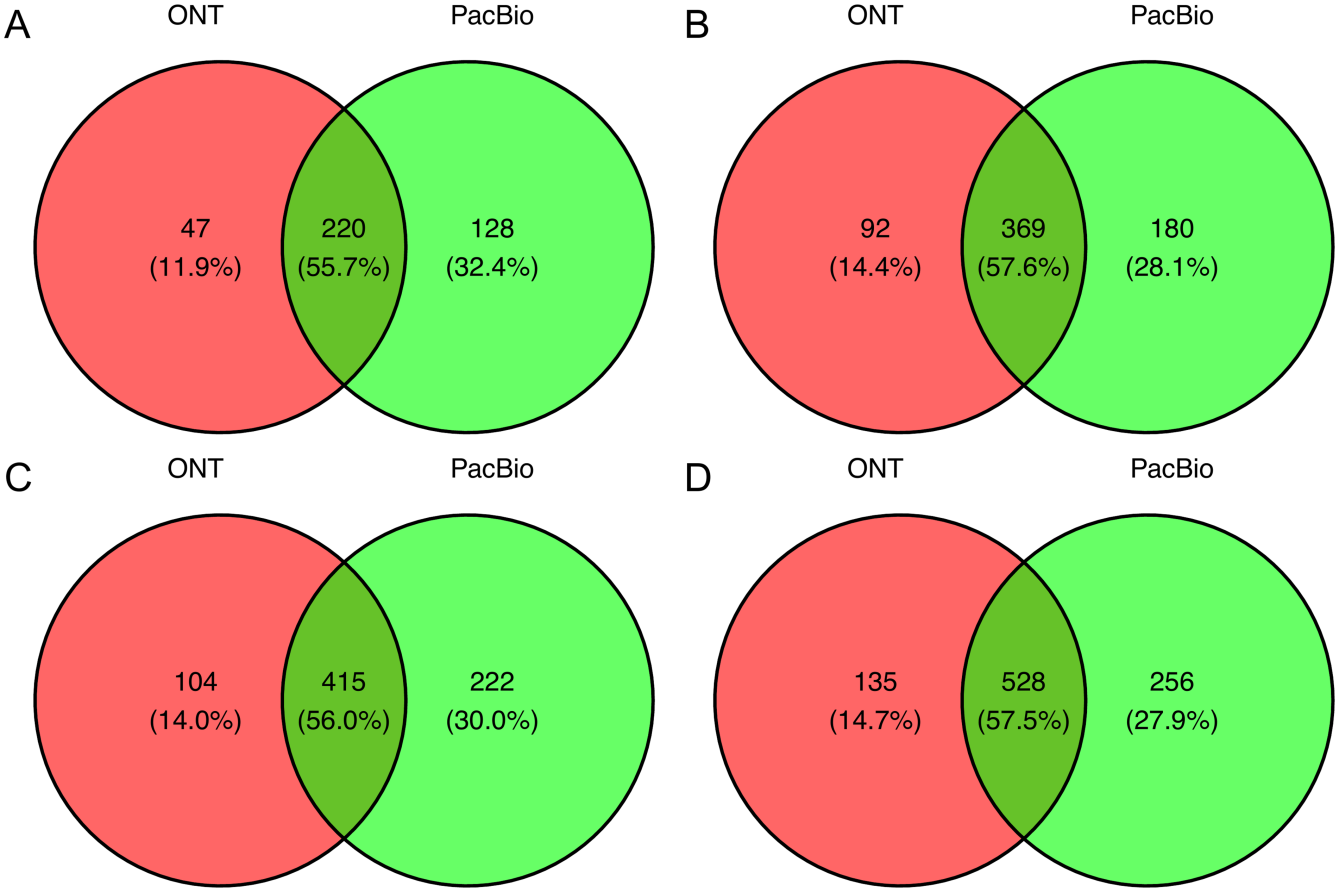
Venn diagram showing common and unique bacterial species between PacBio and ONT sequencing platforms. The comparison was conducted across groups with read counts of **(A)** 10k, **(B)** 20k, **(C)** 25k, and **(D)** 35k.

After evaluating the relationship between read depth and the number of identified species, we assessed the overall overlap in species detected by each sequencing technology (Figure 2). Approximately half of the total identified species were detected by both technologies, with 55.7% overlap at 10,000 reads, increasing to 57.6% at 20,000, 56% at 25,000, and 57.5% at 35,000 reads. Additionally, PacBio identified a greater number of unique species compared to ONT, detecting 20.5% more at 10,000 reads, 13.7% more at 20,000, 16% more at 25,000, and 13.2% more at 35,000 reads, suggesting its higher sensitivity toward certain bacterial taxa. When analyzing unique species in the metataxonomics community for each sequencing technology, the observed differences are mainly attributed to bacteria whose proportion usually does not exceed 0.5% of the total read count and only in rare cases reaches 2% (Supplementary Table S4).

### Diversity analysis and sequencing technology clustering

**Figure 3:**
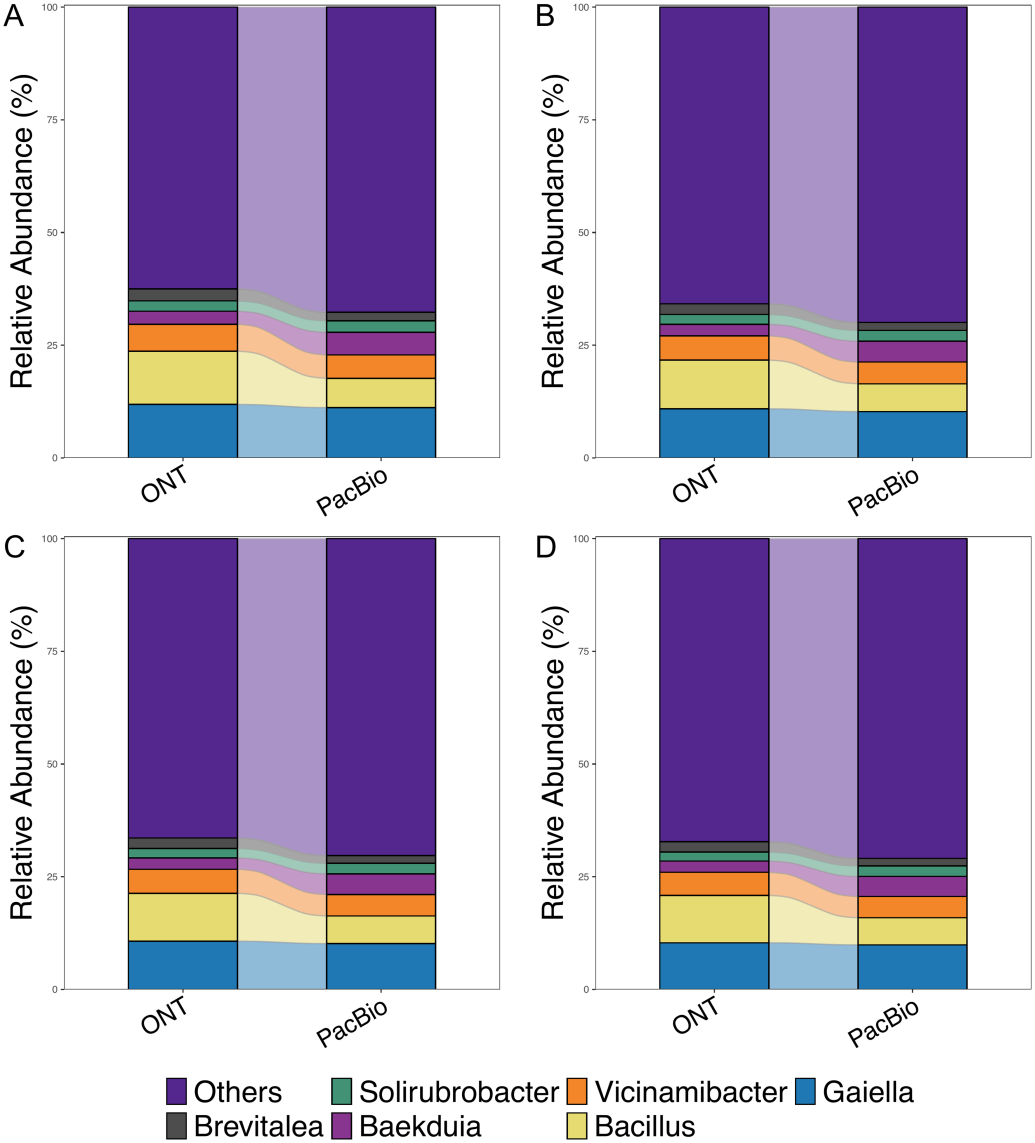
Relative abundance of bacteria at the genus level for ONT and PacBio. The horizontal axis represents the sequencing technology groups, while the vertical axis shows the relative abundance (%). Comparisons were made across groups with read counts of **(A)** 10k, **(B)** 20k, (C) 25k, and **(D)** 35k.

After evaluating the overlap of species identified by both technologies, we analyzed the alpha diversity for each group using the Shannon index to assess species richness in relation to sequencing depth (Supplementary Figure S2). The comparison showed statistically significant differences (*p* < 0.01) between ONT and PacBio data at 10,000 reads (Supplementary Figure S2A). However, with an increase in read numbers to 20,000, 25,000 and 35,000, no statistically significant differences were observed (Supplementary Figures S2B–D). To further investigate the impact of sequencing technology on MC composition, we analyzed beta diversity. Regardless of the read count, samples consistently formed two distinct clusters corresponding to the sequencing technology used (Supplementary Figure S3). However, the application of the Procrustes test for every sequencing depth resulted in *p*-value equal to 0.001 and consequently showed that the structure of MC were similar for both technologies. This indicates that each platform contributes uniquely to bacterial diversity detection but they both can be used for overall interpretation of MC structure. The top six bacterial genera identified in sequencing data using ONT and PacBio platforms were *Brevitalea, Solirubrobacter, Baekduia, Vicinamibacter, Bacillus and Gaiella* (Figure 3). Notably, these dominant genera remained consistent across all sequencing depths, indicating that increasing the number of reads does not significantly alter the identification of the most abundant taxa.

### Illumina vs. ONT: differences in sensitivity and taxonomic profiling

**Figure 4:**
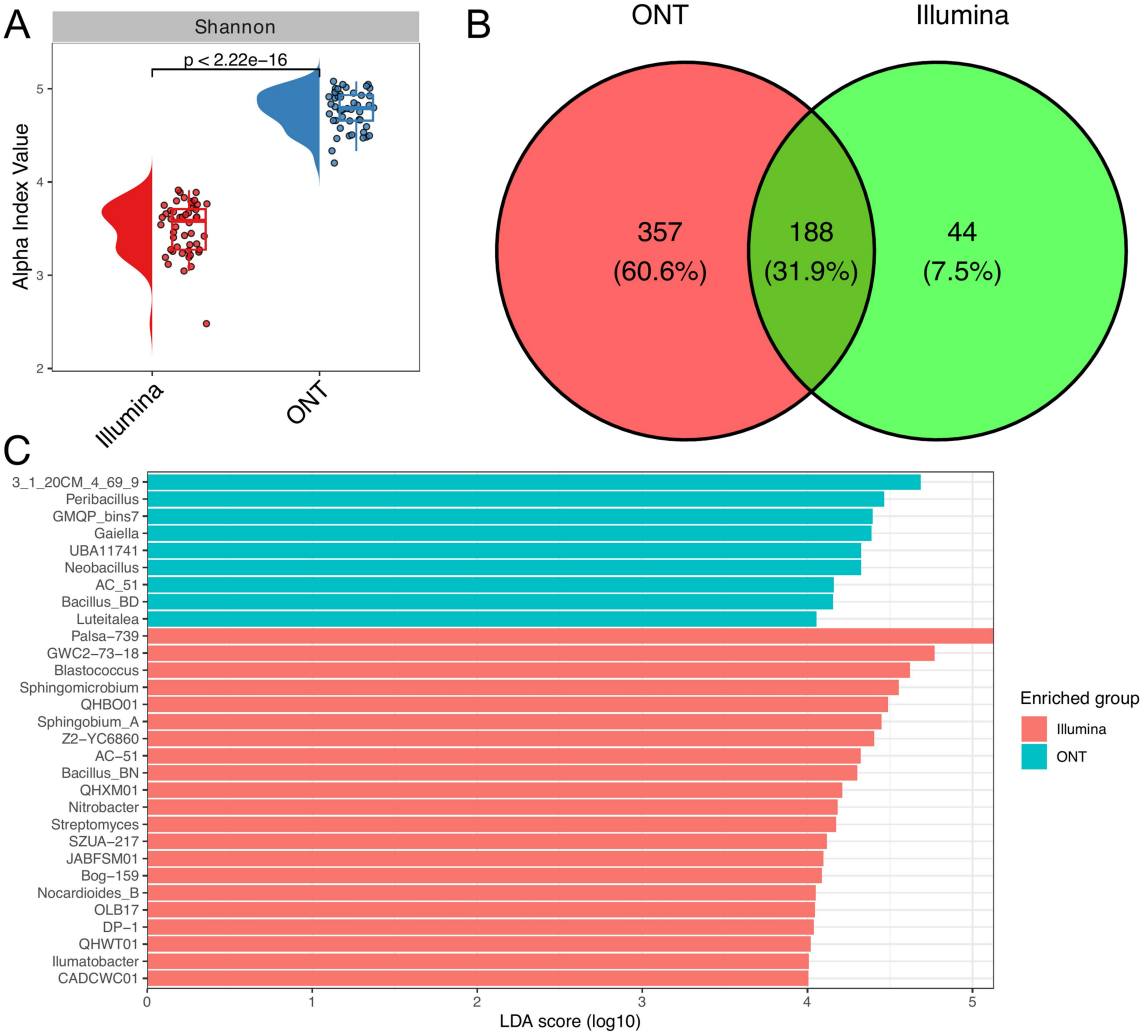
Comparative taxonomic analysis using Illumina and ONT. Raincloud plot of the alpha diversity index (Shannon) across different sequencing technologies **(A)**. The Wilcoxon rank-sum test was applied to determine statistical differences between experimental groups (*n* = 48 for each technology). **(B)** Venn diagram demonstrates the overlap in identified genera between different sequencing technologies. **(C)** Linear discriminant analysis (LDA) effect size was used to determine signature genera across sequencing technologies. The barplot illustrates genera with LDA score greater than 4.

Our comparative analysis of 16S rRNA gene sequencing on Illumina and ONT revealed distinct differences in the sensitivities between platforms. Despite a similar read count, a notable variance was observed in the Shannon index (Figure 4A). In total, Illumina reads accounted for identification of 232 genera, while ONT detected 545. The Venn diagram shows that 188 genera (31.9%) were shared between both platforms (Figure 4B). Furthermore, the results indicate that taxonomic identification from Illumina reads is skewed toward *Actinomycetes* and related bacteria, while ONT reads detect relatively more *Bacilli* bacteria (Figure 4C).

### PacBio vs. Illumina: comparative analysis of V3–V4 and V4 regions

**Figure 5:**
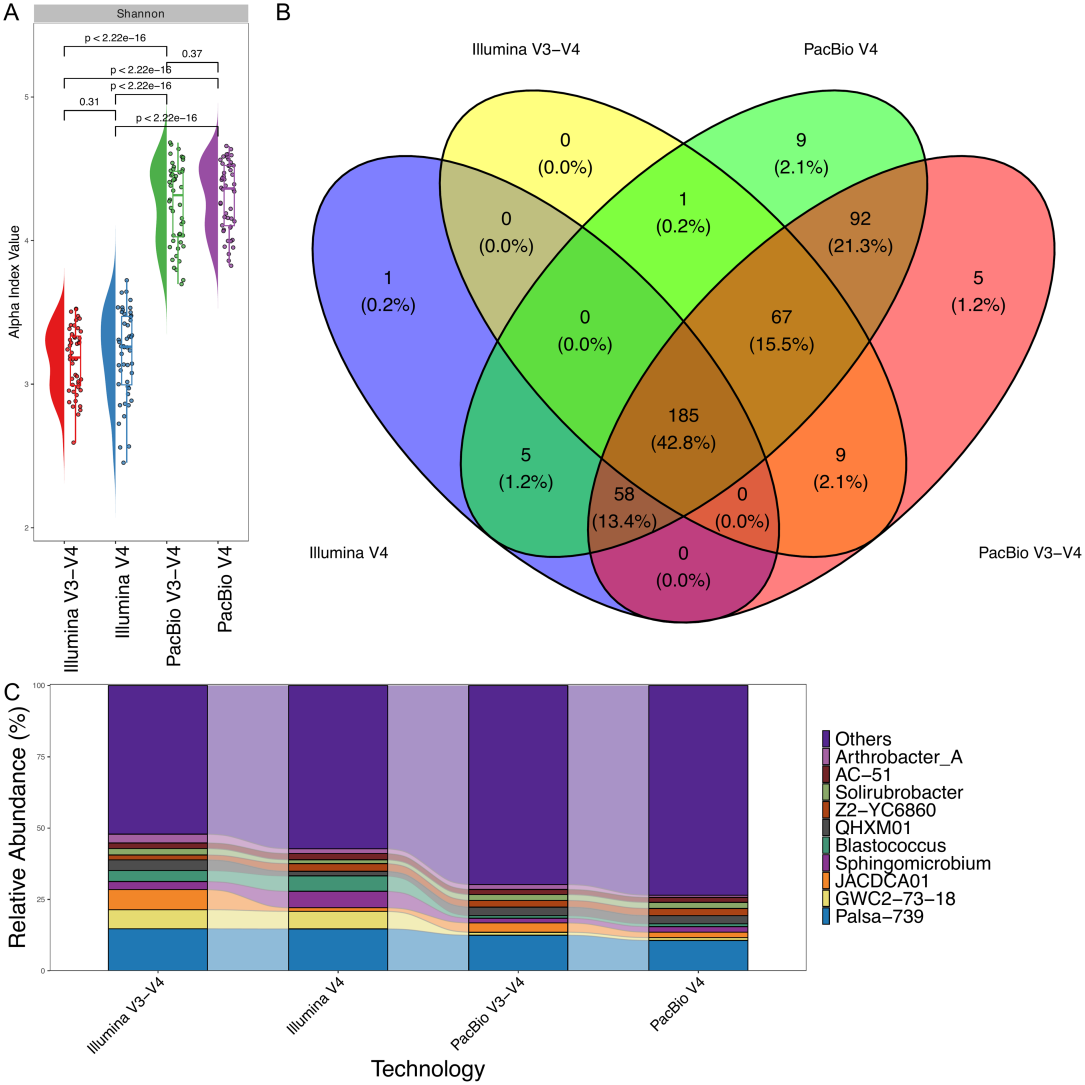
Taxonomic classification comparison for V4 and V3–V4 regions between PacBio and Illumina sequencing platforms (*n* = 48 for each technology). **(A)** Shannon alpha diversity, indicating differences in diversity captured by each platform. The Wilcoxon rank-sum test was applied to determine statistical differences between them. **(B)** Venn diagram showing the overlap in bacterial genera identified by PacBio and Illumina. **(C)** Relative abundance of bacterial genera, comparing the taxonomic composition detected by both technologies.

To ensure comparability between sequencing platforms and minimize the influence of differences in read count and the 16S rRNA region analyzed, PacBio reads were trimmed to the V3–V4 and V4 regions to align with Illumina data (Supplementary Table S1). After trimming, only 18,000 reads per sample on average were retained for analysis. Identical bioinformatic pipelines were then employed to analyze sequence data from both platforms. The analysis of the V3–V4 region on the Illumina platform provided slightly greater insight into the bacterial diversity of soil samples compared to the V4 region analyzed on the same platform (Figure 5A). However, when comparing the same regions sequenced using PacBio technology, the results showed a statistically higher number of bacterial genera identified in the soil samples, highlighting the enhanced resolution of PacBio for full-length and targeted 16S rRNA analysis (Figure 5A).

As illustrated in Figure 5B, 61.2% of bacterial genera were identified by both PacBio and Illumina platforms for the V3–V4 and V4 regions. However, PacBio sequencing detected a significantly broader range of unique genera (24.6% for the V3–V4 and V4 regions), whereas Illumina data has nothing unique for the V3–V4 and V4 regions. This highlights the higher resolution and broader taxonomic coverage of PacBio sequencing. Figure 5C further supports these findings, demonstrating that PacBio sequencing detects a greater number of minor bacterial species compared to Illumina. This broader detection contributes to the higher alpha diversity observed with the PacBio platform, underscoring its enhanced capacity for capturing low-abundance taxa.

PacBio sequencing detects a greater number of minor bacterial species, contributing to the increased alpha diversity observed with this platform (Figure 5C). However, certain genera show notable differences between the sequencing technologies. For example, the relative abundance of *Blastococcus, Sphingomicrobium,* and *GWC2-73-18* is significantly higher in Illumina sequencing compared to PacBio.

Additionally, both Illumina and PacBio technologies show specific trends based on the analyzed regions. When analyzing the V3–V4 region, both platforms detect significantly higher levels of *JACDCA01* bacteria. Similarly, analysis of the V4 region reveals significantly more bacteria from the genus *Sphingomicrobium* with Illumina technologies. Notably, across all regions analyzed by both platforms, the genus *Palsa-739* (*Actinobacteria*) exhibits a nearly identical level of representation, highlighting the consistency of these technologies in detecting certain bacterial groups.

### Evaluating the sensitivity of ONT, PacBio, and Illumina for 16S rRNA gene sequencing

Our comparative analysis of 16S rRNA gene sequencing using ONT, PacBio, and Illumina platforms revealed significant differences in their sensitivities. To ensure consistency and accuracy, sequencing data were preprocessed using platform-specific tools. Emu was employed for ONT and PacBio full-length gene sequences, while the DADA2 pipeline with the GTDB database was used for taxonomic classification at the genus level for PacBio V4/V3–V4 and Illumina regions.

**Figure 6:**
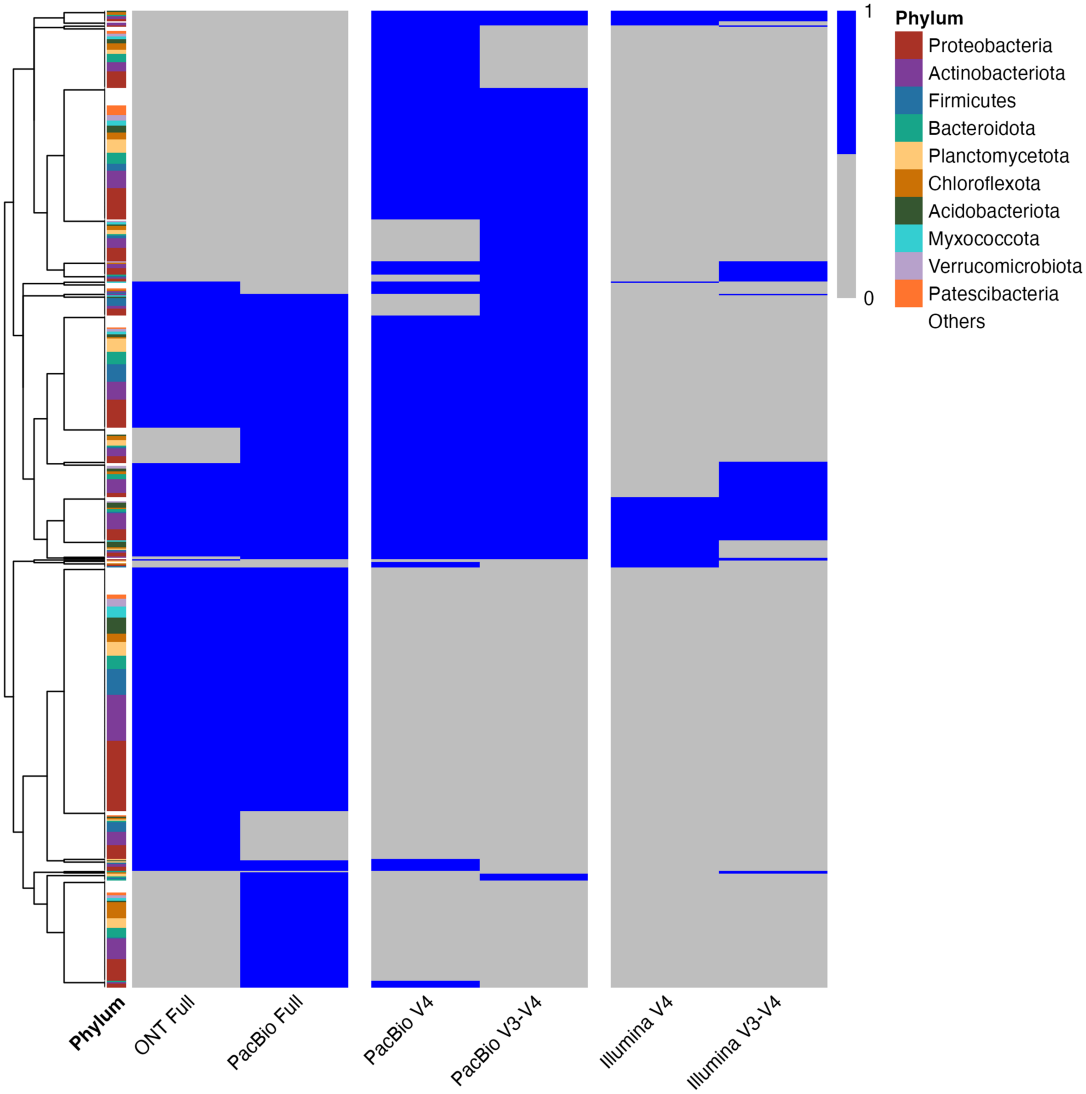
Heatmap depicting the presence/absence of microbial genera across different sequencing technologies. The heatmap illustrates microbial genera defined by the GTDB database. Blue indicates presence, while gray represents absence. The y-axis lists GTDB-annotated microbial genera, and the x-axis denotes different sequencing technologies. The left color bar indicates taxonomic classification at the phylum level. Clustering was performed using Euclidean distance and complete linkage.

For the analysis, 20,000 reads per sample were selected for ONT and PacBio, while 18,000 reads were used for PacBio and Illumina regions. This read depth was chosen to ensure comparability with previously presented results. The analysis showed that ONT and PacBio provide comparable bacterial diversity assessments (Figure 6 and Supplementary Table S5). In contrast, analysis of the V3–V4 and V4 regions using Illumina demonstrated significantly lower taxonomic diversity compared to ONT and PacBio. These findings underscore the importance of selecting an appropriate sequencing platform to achieve the desired level of taxonomic resolution and address specific research objectives.

### Consistency of group separation across sequencing technologies

**Figure 7:**
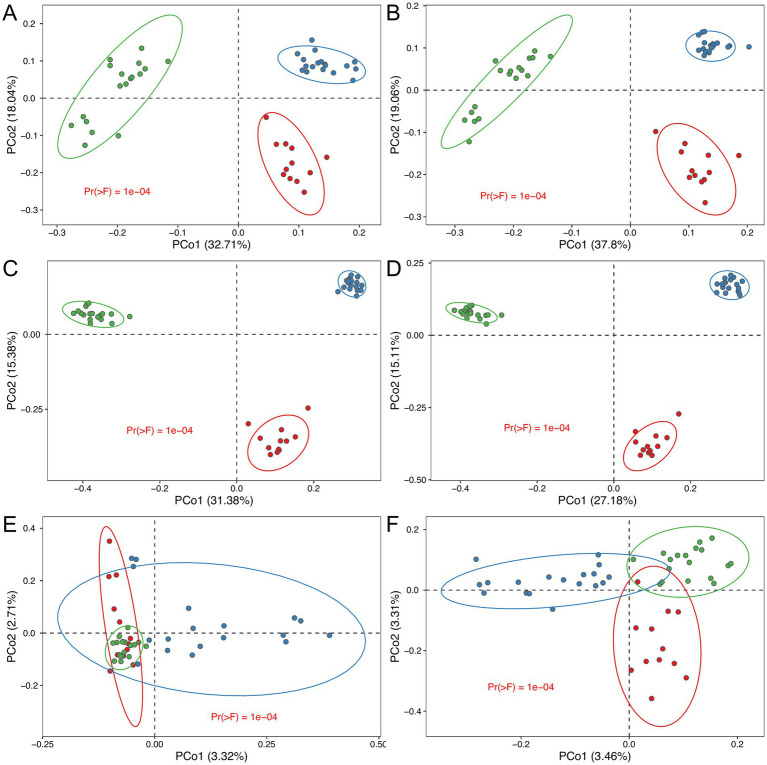
Multidimensional scaling (MDS) biplot, providing a two-dimensional visualization of multidimensional taxonomic profiles derived from various sequencing platforms and 16S rRNA regions. The plots illustrate distinct clustering patterns consistent across sequencing technologies, reflecting group-level differences in microbial community composition: **(A)** ONT full-length; **(B)** PacBio full-length; **(C)** PacBio V4 region; **(D)** PacBio V3–V4 region; **(E)** Illumina V4 region; **(F)** Illumina V3–V4 region. Statistical significance between groups was determined using PERMANOVA (*n* = 12, 18, 18 for black leached, permafrost meadow black and gray soil type respectively).

Our analysis demonstrates that the choice of sequencing technology does not significantly impact the ability to detect group separation within the experimental dataset. Regardless of whether ONT, PacBio, or Illumina platforms were used, all sequencing technologies consistently identified distinct bacterial community compositions corresponding to predefined experimental groups (Figure 7). This consistency is evident from the clustering patterns observed in the principal coordinate analysis (PCoA) plots and further confirmed by permutational multivariate analysis of variance (PERMANOVA), which showed statistically significant group separation (*p* < 0.05) across all platforms and regions analyzed, with the exception of the V4 region (Figures 7; Supplementary Figure S4).

The use of ONT (Figure 7A), PacBio (Figure 7B), and Illumina (V3–V4) (Figure 7F) technologies enabled the clear separation of three distinct soil groups based on their metataxonomics composition. This separation was consistently observed across these platforms, reflecting their robustness in identifying group-level differences in MC.

However, when analyzing beta diversity in the V4 region (Illumina), such distinct group separation was not observed. No statistically significant differences were detected between the groups, highlighting a limitation in the resolution of this approach. This limitation is further supported by alpha diversity analysis (Supplementary Figure S4), which shows that for the V4 region (Illumina), the gray soil group does not significantly differ from the permafrost meadow black soil group (Supplementary Figure S4E, *p* = 0.79). These findings suggest that while all sequencing technologies are effective at detecting group-level differences, their resolution and ability to distinguish between closely related groups may vary depending on the sequencing platform and the targeted 16S rRNA region.

## Discussion

Soils are complex ecosystems characterized by immense variability in ecological properties and MC structure on both global and local scales (Fierer, 2017). Numerous studies have documented the intricate relationships between microbial traits and soil characteristics, with most focusing on individual parameters such as pH, temperature, vegetation, texture, soil moisture content, nitrogen levels, organic matter, or contaminants (He et al., 2006; Ge et al., 2008; Fierer et al., 2009; Lauber et al., 2009; Griffiths et al., 2011; Lanzen et al., 2016; Liu et al., 2016; Zhou et al., 2016). These studies have provided valuable insights into specific interactions, yet they often fail to capture the multifaceted nature of soil ecosystems.

The relevance of the study lies in the growing need to accurately profile soil microbiomes, which play a crucial role in ecosystem functioning and agricultural productivity. Our study’s novelty stems from the detailed comparison of PacBio, ONT and Illumina sequencing technologies at varying read depths, highlighting their strengths and limitations in detecting bacterial species in soil samples. To enhance the robustness of our analysis, we included three biological replicates per soil sample and retained only taxa consistently detected across replicates. This strategy reduced random variation and improved the reliability of diversity estimates, allowing for more confident interpretation of platform performance.

Recent advancements in sequencing technologies, including PacBio, ONT, and Illumina platforms, have provided unprecedented opportunities to explore soil microbiomes in detail. These tools enable researchers to link microbial diversity with soil properties more comprehensively, offering insights into the complex mechanisms that govern MC composition and function. Such integrative approaches are crucial for advancing our understanding of the ecological processes underpinning soil ecosystems and their responses to environmental changes.

Alpha diversity analysis applying the Shannon index, a widely used metric accounting both species richness and evenness within a sample (Roswell et al., 2021), showed statistically significant differences between ONT and PacBio data at 10,000 reads, but not at 20,000, 25,000 and 35,000 reads. The results indicate that while low read depths may show significant differences in diversity metrics, due to their sensitivity to sequencing effort (Schloss, 2024), increasing the read depth mitigates this effect, leading to more comparable results between sequencing technologies. Such results at low sequencing depth can also be caused by randomness during rarefaction of samples’ data. This aligns with the rarefaction curve analysis, which demonstrated that bacterial diversity in the samples reaches a plateau after 35,000 reads, indicating sufficient taxonomic coverage. These findings emphasize the importance of selecting an optimal read count for capturing the full scope of microbial diversity and are consistent with previously published data (Hussein et al., 2017; Mysara et al., 2017). Higher efficiency of PacBio at lower read depth can be attributed to its longer read lengths and higher accuracy due to employment of circular consensus sequencing technology which is based on the generation of multiple reads of the same DNA molecule, thereby reducing random errors (Wang et al., 2019; Kim et al., 2024). PacBio’s high sensitivity in microbiome analysis has been previously demonstrated, showing 100% specificity and sensitivity in taxonomic classification of a mock community with 20 bacterial species (Earl et al., 2018). ONT, on the other hand, offers advantages such as portability and cost-efficiency, but its sensitivity and accuracy are currently considered inferior to PacBio (Straub et al., 2020).

Despite technological differences, overall trends in alpha diversity remained consistent across sequencing platforms. Long-read technologies such as PacBio and ONT provide better coverage of hypervariable regions within the 16S rRNA gene, thereby improving taxonomic classification (Nygaard et al., 2020; Santos et al., 2020; Szoboszlay et al., 2023; Buetas et al., 2024). However, there are trade-offs to consider when selecting a platform for microbiome analysis: while Illumina offers a more established and standardized bioinformatics pipeline, PacBio and ONT generate longer reads that can enhance resolution and accuracy in profiling bacterial communities. Moreover, current long-read classification tools, such as Emu, lack explicit chimera detection modules, which may affect data quality in complex communities. This highlights the need for further development of chimera-aware algorithms tailored to long-read 16S rRNA data. Other tools, such as VSEARSH (Rognes et al., 2016), can perform *de novo* chimera detection. However, in this study the chimera removal step is absent. Instead of this in order to make our taxonomic profiling more accurate we applied filtering of observed OTUs by their presence in biological repeats. In the broader context, ongoing improvements in basecalling algorithms (e.g., Dorado super mode), error-correction tools (such as Medaka and NanoCLUST), and new taxonomic classifiers specifically tailored for long, noisy reads (e.g., Emu) are helping to improve the reliability of ONT-based microbial profiling (Zhang G. et al., 2024; Zhang T. et al., 2024).

Beta diversity analysis revealed that samples formed two distinct clusters corresponding to the type of sequencing technology, indicating that each technology contributes uniquely to the detection of bacterial diversity and influences the outcomes of MC analysis. This distinct clustering suggests that PacBio and ONT may have different biases in detecting certain bacterial taxa, underscoring the importance of considering the choice of sequencing technology when interpreting MC data (Kim et al., 2024). Analysis of the overlap and uniqueness of species identified by each technology revealed that more than 57% of the total species were observed applying both technologies, indicating their complementarity in detecting bacterial diversity. This complementarity suggests that using both technologies in tandem could provide a more comprehensive view of the soil microbiome, a finding supported by previous research (Kim et al., 2024).

Both ONT and PacBio demonstrated a similar efficiency in identifying the top 6 bacterial genera. This suggests that key bacterial genera in the samples can be reliably detected even at lower sequencing depths, while additional sequencing primarily contributes to identifying low-abundance taxa.

Differences in the number of bacterial species detected in soil samples between PacBio and ONT may be attributed to several factors related to the technological characteristics of these platforms as well as bioinformatics data processing. A total of 0.52% of reads from PacBio and 16.7% of reads from ONT failed filtering, indicating the lower accuracy of ONT. This lower accuracy can lead to errors in species identification, particularly in homopolymeric regions or when analyzing closely related sequences. In addition to differences in read accuracy and filtering rates, our results suggest that PacBio may be more effective than ONT in detecting low-abundance taxa in soil samples. This discrepancy could arise from differences in bioinformatic processing pipelines. For ONT, error correction algorithms often remove low-frequency variants, mistaking them for sequencing artifacts. In contrast, PacBio’s CCS provides high per-read accuracy (>99%) without aggressive filtering, thereby preserving rare sequence variants (Johnson et al., 2019). Furthermore, soil microbial communities include many high-GC-content organisms, which may be systematically underrepresented in ONT datasets due to known biases in basecalling and pore-level performance. PacBio appears to be less affected by GC bias, which may further enhance its ability to recover rare taxa from complex environments like soil. Recent developments in ONT bioinformatics, particularly error correction tools based on machine learning and deep learning frameworks, are further enhancing read accuracy and taxonomic resolution. For example, DeChat, a novel tool that incorporates repeat- and haplotype-aware models, significantly improves the correction of Nanopore reads in complex genomic regions (Liu et al., 2024). Although not directly applied in our current 16S rRNA amplicon study, such methods hold promise for future integration into long-read metataxonomic pipelines, especially when dealing with highly diverse or repetitive microbial genomes. However, one of the inherent limitations of 16S rRNA gene sequencing lies in its inability to reliably distinguish between closely related bacterial species that share high sequence similarity in this gene. For instance, *Veillonella rogosae* and *V. parvula* exhibit 98% homology in their 16S rRNA gene sequences, which often leads to misidentification (Arif et al., 2008). This limitation underscores the need for whole-metagenome sequencing approaches to achieve more accurate species-level identification.

The Illumina MiSeq platform, with a read length limitation of up to 600 bp, does not allow for full-length 16S rRNA gene sequencing. Instead, its hypervariable regions can be used individually or in combination to evaluate bacterial community structures. Previous studies have demonstrated that the choice of primers and targeted regions significantly influences microbiome profiling results. These factors should therefore be carefully considered during the sequencing (Klindworth et al., 2013; Albertsen et al., 2015; Tremblay et al., 2015; Fouhy et al., 2016; Rintala et al., 2017; Fuks et al., 2018; Zhang et al., 2018).

A substantial body of research has explored the impact of primer selection on microbiome community profiling. For example, studies have shown that the analysis of human microbiomes and microbial alpha diversity in fecal samples varies depending on whether the V3–V4 or V4–V5 region is targeted (Rintala et al., 2017). Conversely, other studies have reported minimal differences in community profiling when regions such as V1–V3, V3–V4, or V4 are used within the same sample type. Comparable research conducted on environmental samples, such as water, suggests that the V4 region may be more suitable for precise sequence assignment in the bacterial domain while also offering increased coverage (Zhang et al., 2018).

Albertson et al. reported differences in the distribution of bacterial taxa when targeting V1–V3, V3–V4, and V4 regions, yet observed similar alpha diversity values across these regions (Albertsen et al., 2015). However, gaps remain in our understanding, particularly for highly diverse samples such as those derived from soil. Current studies lack detailed insights into the influence of 16S rRNA regions on bacterial community profiling for both environmental and biological samples. Furthermore, there is an absence of comprehensive analyses that include the use of combined regions such as V1–V3, V3–V4, V4–V5, and V6–V8, which are frequently employed to enhance taxonomic accuracy in amplicon sequencing (Soriano-Lerma et al., 2020).

Future research should address existing gaps by investigating how different 16S rRNA regions and primer combinations influence the profiling of complex and diverse MC (Strokach et al., 2025). This discrepancy underscores the critical role of primer selection in microbiome studies. While region-specific primers, such as those targeting the V4 region, may enhance taxonomic resolution for specific groups (Kozich et al., 2013; Zhou et al., 2024), they risk overlooking minor bacterial populations essential for understanding MC complexity. In contrast, universal primers used for full-length 16S rRNA gene sequencing provide a more comprehensive view of community evenness, making them better suited for studies aiming to capture a broad range of taxa (Schloss et al., 2009; Caporaso et al., 2012). These findings highlight the need to carefully consider primer design and target region selection when planning sequencing-based microbiome studies. Future research should further explore the trade-offs between primer specificity and universality to optimize microbial diversity assessments across different environments (Abellan-Schneyder et al., 2021), ultimately improving the reliability of microbiome analyses and guiding the selection of sequencing strategies for both targeted and high-resolution profiling.

The observed differences in taxonomic profiles between regions sequenced on PacBio and Illumina platforms may be attributed to the primers used during the initial PCR step in library preparation. Primers targeting the full-length 16S rRNA gene, as used in PacBio sequencing, are designed to be more universal and inclusive, allowing for a broader representation of bacterial taxa, including low-abundance groups. In contrast, primers targeting the V4 region, often employed in Illumina sequencing, are more specific to certain bacterial species, which can limit their effectiveness in identifying minor taxa and reduce overall taxonomic resolution (Klindworth et al., 2013; Apprill et al., 2015).

In our analysis, we also observed differences in taxonomic composition depending on the sequencing technology used. Sequencing with the MiSeq platform showed an increased presence of the genera *Peribacillus, Gaiella,* and *Neobacillus*, whereas the ONT platform identified a higher number of taxa belonging to *Palsa-739, Blastococcus, Sphingomicrobium, Sphingobium,* and *Bacillus*. These results are consistent with literature data indicating that the sequencing platform has a significant impact on the relative abundance of taxa. Specifically, MiSeq sequencing tends to detect a higher proportion of *Actinobacteria, Chloroflexi,* and *Gemmatimonadetes*, while the abundance of *Acidobacteria, Bacteroides, Firmicutes, Proteobacteria,* and *Verrucomicrobia* is lower compared to the MinION platform (Stevens et al., 2023). Thus, taxonomic identification based on Illumina reads appears to be biased toward *Actinobacteria* and related groups, whereas ONT enables the detection of a greater number of *Bacilli* representatives.

Our analysis demonstrated a high level of concordance in bacterial composition down to the genus level when using both sequencing platforms (Illumina and PacBio). A comparison of the presence and absence of taxonomically assigned bacteria showed that most genera were detected on both platforms, indicating that the obtained data are comparable and suitable for future genus-level comparisons (Buetas et al., 2024). Similar results, but using ONT, were reported in the study by Matsuo et al., which assessed the efficiency of full-length 16S rRNA gene sequencing with the MinION^™^ technology for analyzing human fecal samples. Their findings also confirmed that different sequencing platforms, particularly Illumina and ONT, provide reproducible results at the genus level (Matsuo et al., 2021).

In summary, all three sequencing platforms (ONT, PacBio, and Illumina) are suitable for determining the composition of MC based on the 16S rRNA gene. In our study, we analyzed three different soil types, and the results of metataxonomics sequencing obtained from all platforms allowed for a clear differentiation between them. However, it is important to note that when using the Illumina technology to analyze only the V4 variable region, no distinct clustering of samples based on soil type was observed. Additionally, each platform exhibits taxonomic biases, which may complicate the comparison of results across studies utilizing different sequencing methods.

## Conclusion

A comparative analysis of the PacBio and ONT platforms for soil microbiome profiling revealed a high degree of similarity in the obtained data, with PacBio demonstrating slightly higher efficiency in identifying low-abundance taxa at lower sequencing depths. Our results highlight the importance of selecting an optimal sequencing depth and considering the specific characteristics of each technology when analyzing MC. We demonstrated that all three platforms (ONT, PacBio, and Illumina) are capable of assessing bacterial diversity in soil samples and distinguishing them into groups based on their origin. Furthermore, the data obtained from ONT are comparable to those from PacBio, indicating that sequencing errors inherent to ONT do not significantly impact the interpretation of well-represented taxa. Due to their long-read capability and the ability to analyze the full-length 16S rRNA gene, both ONT and PacBio offer advantages over Illumina for bacterial diversity assessment. Our study confirms that the choice of sequencing technology can substantially influence microbiome data interpretation. Future research should focus on improving bioinformatics approaches, particularly for the detection of rare taxa, while also accounting for taxonomic biases associated with different sequencing platforms.

## Data Availability

The raw sequencing data obtained using PacBio, ONT and Illumina sequencing were submitted to the NCBI Sequence Read Archive (SRA) and are accessible under the following BioProject identifiers: PRJNA1190309 (for 16S rRNA gene sequencing on ONT), PRJNA1190314 [for 16S rRNA gene sequencing on Pacific Biosciences (PacBio)], PRJNA1190320 and PRJNA1190324 [for V3–V4 and V4 region of the 16S rRNA gene sequencing data on Illumina (MiSeq) respectively].

## Footnotes

^1^ https://github.com/rrwick/Porechop

## Supplementary material

SUPPLEMENTARY FIGURE S1 Rarefaction curves illustrate dependence of Shannon alpha diversity index from the number of reads for ONT **(A,C)** and PacBio **(B,D)**. Pictures **(A,B)** show average value (solid line) and SEM (smoothed area) while pictures **(C,D)** show Shannon indexes for each sample individually.

SUPPLEMENTARY FIGURE S2 Raincloud plots show Shannon alpha diversity index for PacBio and ONT sequencing platforms. The comparison was performed across groups with read counts of **(A)** 10k, **(B)** 20k, **(C)** 25k, **(D)** 35k. The Wilcoxon rank-sum test was applied to determine statistical differences between two sequencing platforms (*n* = 48 for each technology).

SUPPLEMENTARY FIGURE S3 Scatterplots of two-dimensional visualization of multidimensional taxonomic profiles derived from PacBio and ONT sequencing platforms. Statistical significance between groups was determined using PERMANOVA (*n* = 48 for each technology). The comparison was conducted across groups with read counts of **(A)** 10k, **(B)** 20k, **(C)** 25k, **(D)** 35k.

SUPPLEMENTARY FIGURE S4 Raincloud plots of the alpha diversity index (Shannon) across different sequencing technologies and 16S rRNA regions: **(A)** ONT full-length; **(B)** PacBio full-length; **(C)** PacBio V4 region; **(D)** PacBio V3–V4 region; **(E)** Illumina V4 region; **(F)** Illumina V3–V4 region. The Wilcoxon rank-sum test was applied to determine statistical differences between soil types (*n* = 12, 18, 18 for black leached, permafrost meadow black and gray soil respectively).

SUPPLEMENTARY TABLE S1 Number of raw reads obtained for each sequencing technology.

SUPPLEMENTARY TABLE S2 Taxonomic classification of the ZymoBIOMICS Microbial Community Standard for ONT.

SUPPLEMENTARY TABLE S3 Unique bacterial species detected by ONT and PacBio.

SUPPLEMENTARY TABLE S4 Unique bacterial species detected by ONT and PacBio.

SUPPLEMENTARY TABLE S5 Taxonomic classification results across all sequencing technologies.
