# Supplementary figures and images for: Comparative evaluation of sequencing platforms: Pacific Biosciences, Oxford Nanopore Technologies, and Illumina for 16S rRNA-based soil microbiome profiling
Source: Front Microbiol. 2025 Aug 6;16:1633360. doi: 10.3389/fmicb.2025.1633360 (PMC12365774; doi:10.3389/fmicb.2025.1633360)

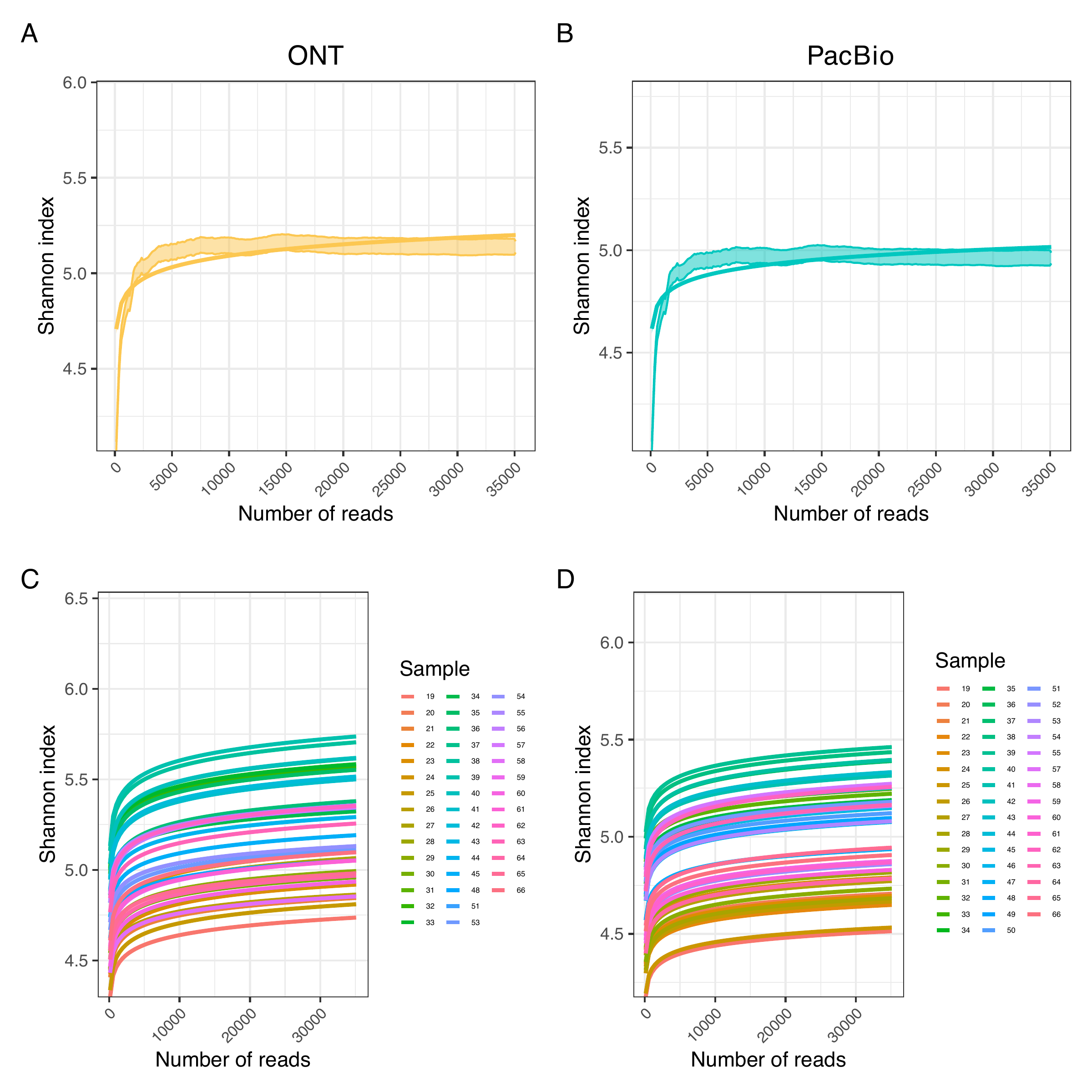

Supplement: SUPPLEMENTARY FIGURE S1 — Rarefaction curves illustrate dependence of Shannon alpha diversity index from the number of reads for ONT (A,C) and PacBio (B,D). Pictures (A,B) show average value (solid line) and SEM (smoothed area) while pictures (C,D) show Shannon indexes for each sample individually. [file Image_1.PNG]

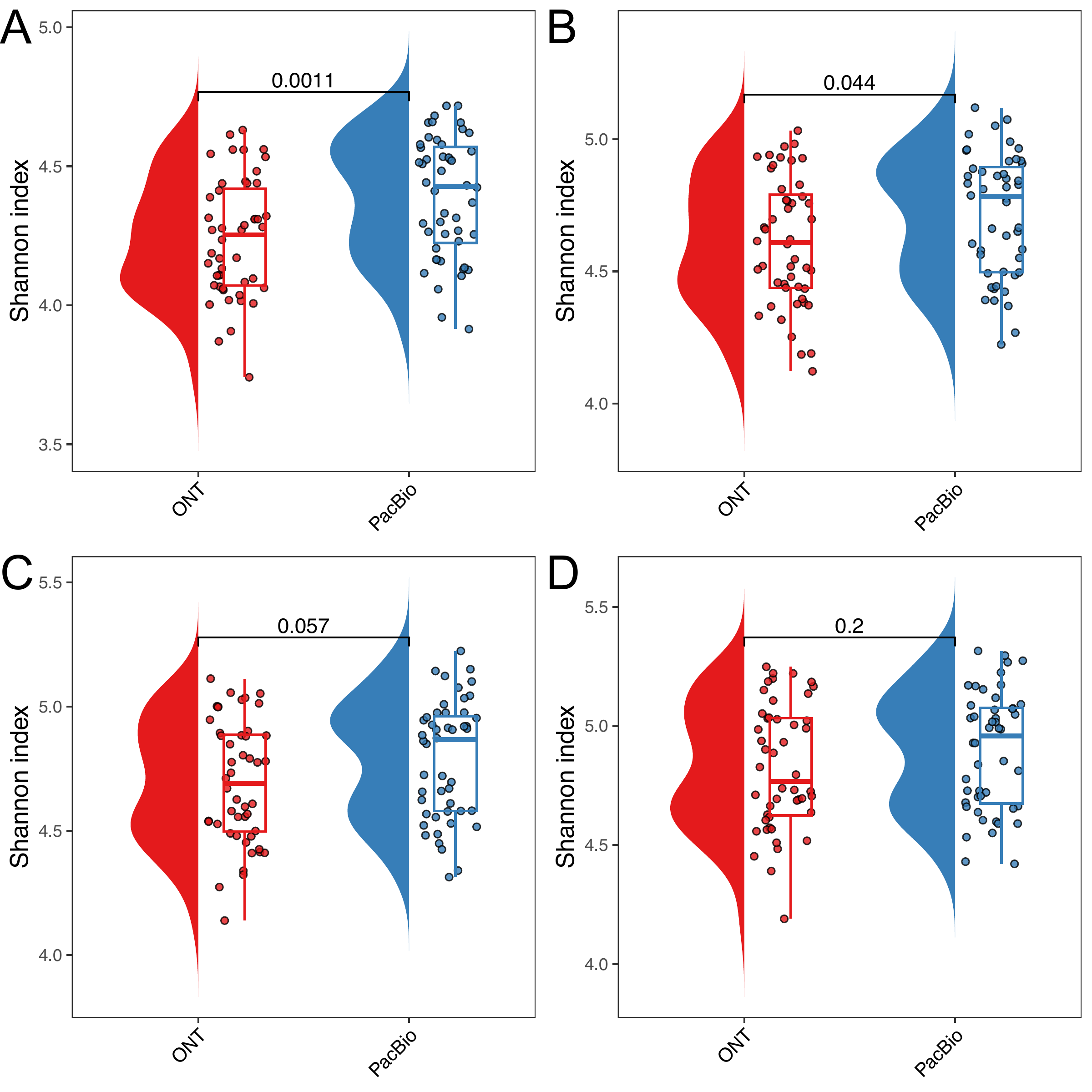

Supplement: SUPPLEMENTARY FIGURE S2 — Raincloud plots show Shannon alpha diversity index for PacBio and ONT sequencing platforms. The comparison was performed across groups with read counts of (A) 10k, (B) 20k, (C) 25k, (D) 35k. The Wilcoxon rank-sum test was applied to determine statistical differences between two sequencing platforms (n = 48 for each technology). [file Image_2.PNG]

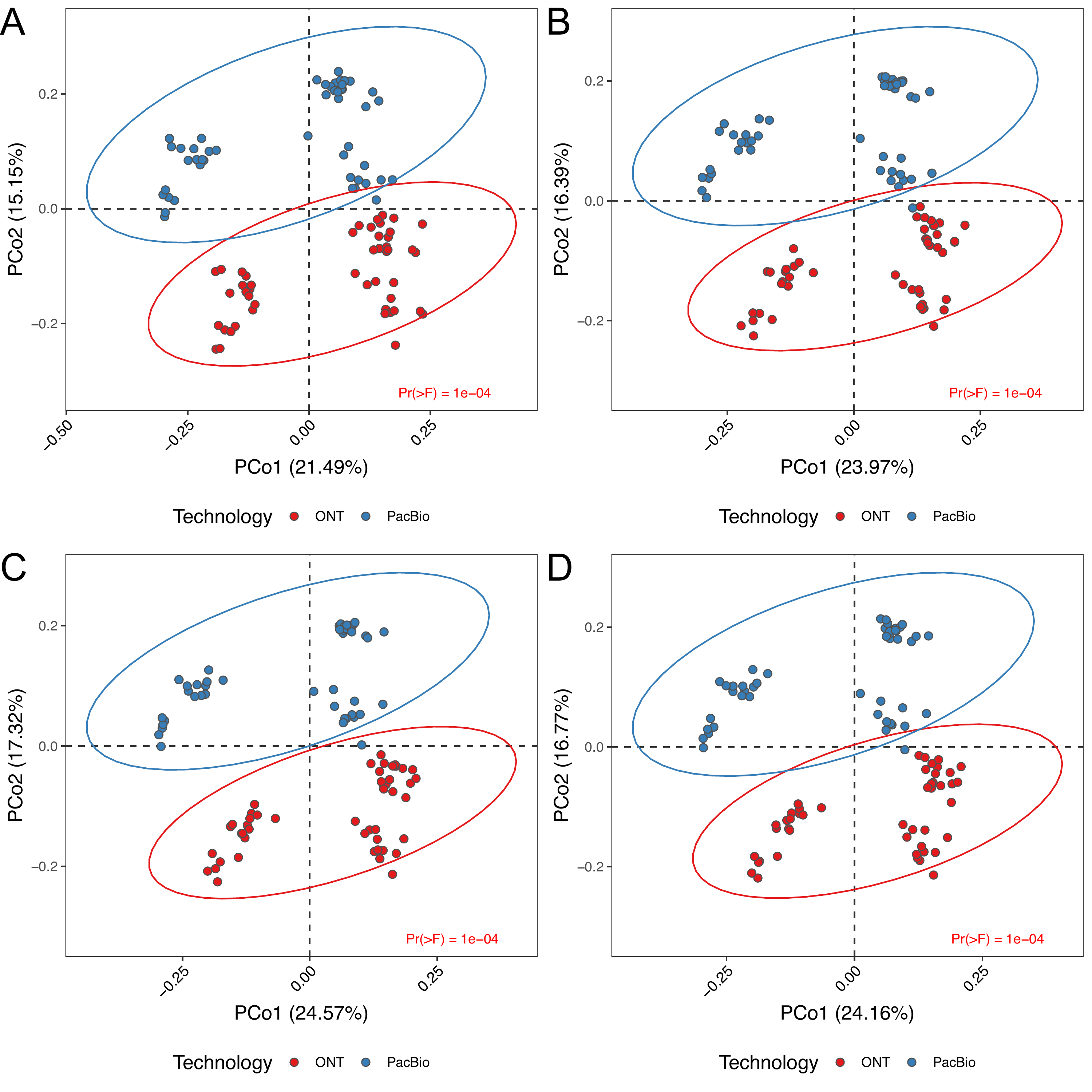

Supplement: SUPPLEMENTARY FIGURE S3 — Scatterplots of two-dimensional visualization of multidimensional taxonomic profiles derived from PacBio and ONT sequencing platforms. Statistical significance between groups was determined using PERMANOVA (n = 48 for each technology). The comparison was conducted across groups with read counts of (A) 10k, (B) 20k, (C) 25k, (D) 35k. [file Image_3.PNG]

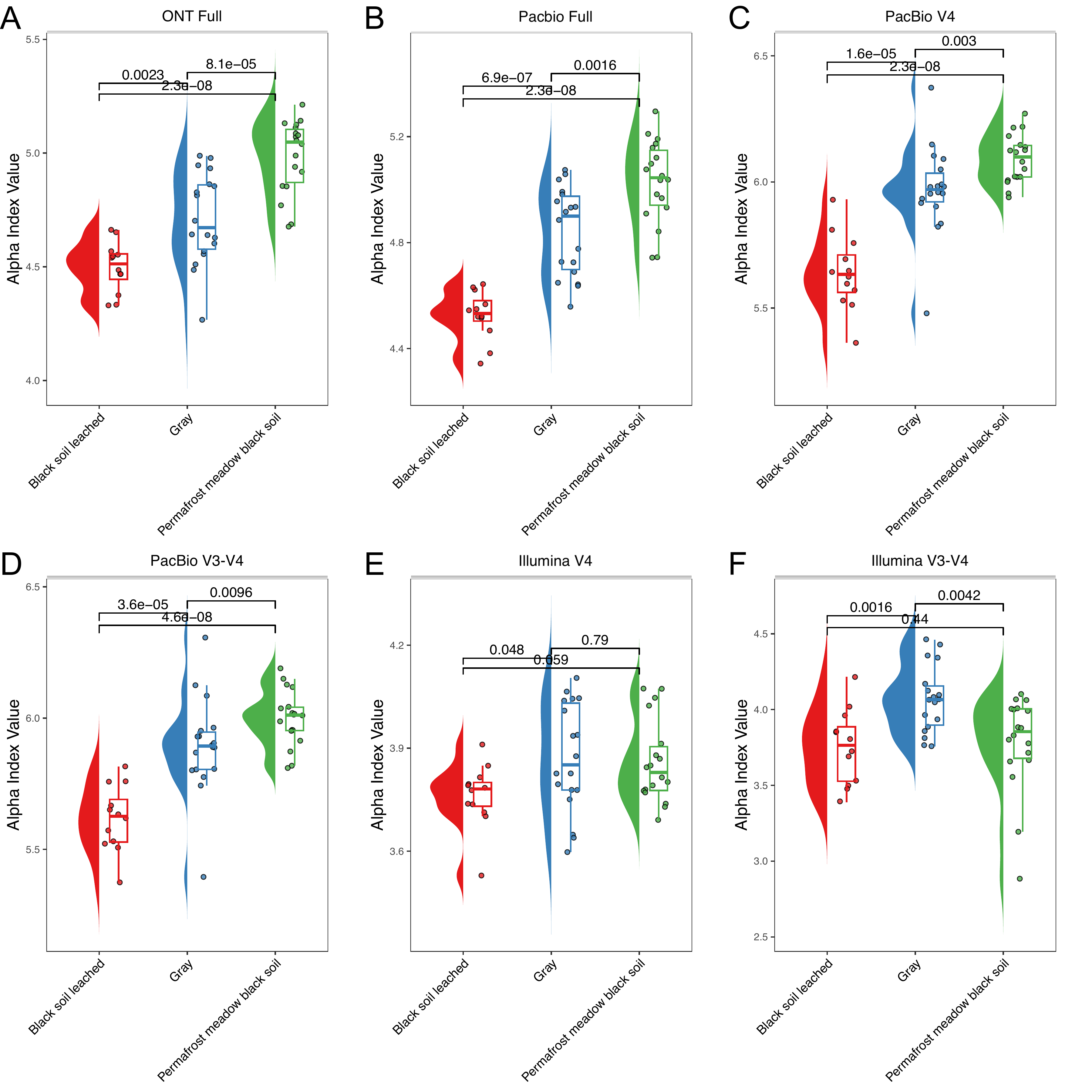

Supplement: SUPPLEMENTARY FIGURE S4 — Raincloud plots of the alpha diversity index (Shannon) across different sequencing technologies and 16S rRNA regions: (A) ONT full-length; (B) PacBio full-length; (C) PacBio V4 region; (D) PacBio V3–V4 region; (E) Illumina V4 region; (F) Illumina V3–V4 region. The Wilcoxon rank-sum test was applied to determine statistical differences between soil types (n = 12, 18, 18 for black leached, permafrost meadow black and gray soil respectively). [file Image_4.PNG]
